# A Novel Mechanism of Immunoproteasome Regulation via miR-369-3p in Intestinal Inflammatory Response

**DOI:** 10.3390/ijms232213771

**Published:** 2022-11-09

**Authors:** Viviana Scalavino, Emanuele Piccinno, Anna Maria Valentini, Mauro Mastronardi, Raffaele Armentano, Gianluigi Giannelli, Grazia Serino

**Affiliations:** National Institute of Gastroenterology “S. de Bellis”, IRCCS Research Hospital, 70013 Castellana Grotte, Bari, Italy; viviana.scalavino@irccsdebellis.it (V.S.); emanuele.piccinno@irccsdebellis.it (E.P.); am.valentini@irccsdebellis.it (A.M.V.); mauro.mastronardi@irccsdebellis.it (M.M.); raffaele.armentano@irccsdebellis.it (R.A.); gianluigi.giannelli@irccsdebellis.it (G.G.)

**Keywords:** miRNAs, miR-369-3p, IBD, immunoproteasome, PSMB9

## Abstract

The immunoproteasome is a multi-catalytic protein complex expressed in hematopoietic cells. Increased expression of immuno-subunits followed by increased proteasome activities is associated with the pathogenesis of IBD. Therefore, the identification of molecules that could inhibit the activities of this complex has been widely studied. microRNAs are small molecules of non-coding RNA that regulate the expression of target genes. Our purpose was to demonstrate that miR-369-3p is able to reduce the expression of the PSMB9 subunit and consequently modulate the catalytic activities of immunoproteasome. After bioinformatics prediction of the gene target of miR-369-3p, we validated its modulation on PSMB9 expression in the RAW264.7 cell line in vitro. We also found that miR-369-3p indirectly reduced the expression of other immunoproteasome subunits and that this regulation reduced the catalytic functions of the immunoproteasome. Increased levels of PSMB9 were observed in colon samples of acute IBD patients compared to the remission IBD group and control group. Our data suggest that miR-369-3p may be a future alternative therapeutic approach to several compounds currently used for the treatment of inflammatory disorders including IBD.

## 1. Introduction

Macrophages are a class of immune phagocytic cells that are mainly involved in the innate immune response and in tissue development and homeostasis [1,2]. During immune response, macrophages are one of the key cells that recognize and process host pathogens and foreign agents that are able to produce several pro-inflammatory cytokines and activate other immune system cells [3]. When homeostasis is perturbed, the pool of macrophages undergoes significant changes that lead to an altered inflammatory response. An accumulation of macrophages is detected in patients affected by inflammatory bowel diseases (IBD) such as Crohn’s disease (CD) and ulcerative colitis (UC) [4,5,6].

Proteasomes are a multi-protein complex constitutively expressed in all eukaryote cells. The 26S proteasome is a multi-catalytic proteinase responsible for the degradation of intercellular damage and poly-ubiquitinated proteins. It is structurally composed of a 20S core with catalytic activities and 19S regulatory complexes [7,8]. The core of proteasome is constituted by four heptameric rings unified to form a cylindrical structure: the two inner rings formed by β-subunits, including β1, β2, and β5, which have catalytic activities, and the two outer α-rings that permit interaction with 19S regulatory complexes [8,9].

Stimulation with various pro-inflammatory cytokines such as TNF-α and INF-γ, as well as TLRs receptor ligands including LPS, induces the replacement of the constitutive proteasome β-subunits with βi-subunits forming the immunoproteasome (iP) [10,11,12,13,14]. Specifically, the β1, β2, and β5 subunits are substituted by β1i, β2i, and β5i also known as Proteasome 20S Subunit Beta (PSMB) 9 (low-molecular-weight protein 2, LMP2), PSMB10 (multi-catalytic endopeptidase complex-like-1, MECL-1) and PSMB8 (low-molecular-weight protein 7, LMP7), respectively. Each βi-subunit plays a specific proteolytic activity, such as a caspase-like activity by β1i, trypsin-like activity by β2i, and chymotrypsin-like activity by β5i [8,9]. The proteolytic core complex requires the association with regulatory complexes that permit the access of substrates to the lumen of the core 20S and guide the iP cleavage activities. The 19S regulatory complex, required in constitutive proteasomes, also known as PA700, is substituted by an 11S regulatory complex, namely PA28, composed by the two subunits Proteasome Activator Subunit 1 (PA28α) and Proteasome Activator Subunit 2 (PA28β). Its function is to generate peptide fragments of pathogen-derived proteins, which are subsequently submitted to major histocompatibility complex (MHC) class I [13,15].

iP is expressed in macrophage cells and plays a key role as a regulator of their functions and the inflammatory process [13,16,17]. The augmented expression of immuno-subunits with a consequent increase in proteasomal activities has been demonstrated to be involved in the progressive inflammation in IBD [18,19,20]. Several studies have demonstrated that in vitro and in vivo treatments with iP inhibitors attenuate the experimental colitis [21,22,23,24].

microRNAs (miRNAs) are small molecules of non-coding RNA, 20–22 nucleotides in length, that regulate gene expression at the post-transcriptional level. miRNAs are implicated in several biological processes, including inflammation [25,26,27,28,29]. Our previous works have shown that miR-369-3p could be a good candidate for the treatment of chronic inflammation. In fact, it shows efficient modulation of C/EBP-β and NOS2 expression, involved in the regulation of pro-inflammatory cytokines production and the activation of NF-kB signaling pathways [30,31].

The aim of the present study was to investigate the effect of miR-369-3p on immunoproteasome expression. We demonstrated that in macrophages, under inflammatory conditions, miR-369-3p is also able to decrease the expression of PSMB9/LMP2. This reduction consequently decreases the expression of the other immunoproteasome subunits, modulating the formation of the complex itself. These findings further validate the anti-inflammatory effect of miR-369-3p.

## 2. Results

### 2.1. In Silico Analysis of miR-369-3p Target Genes

**Figure 1:**
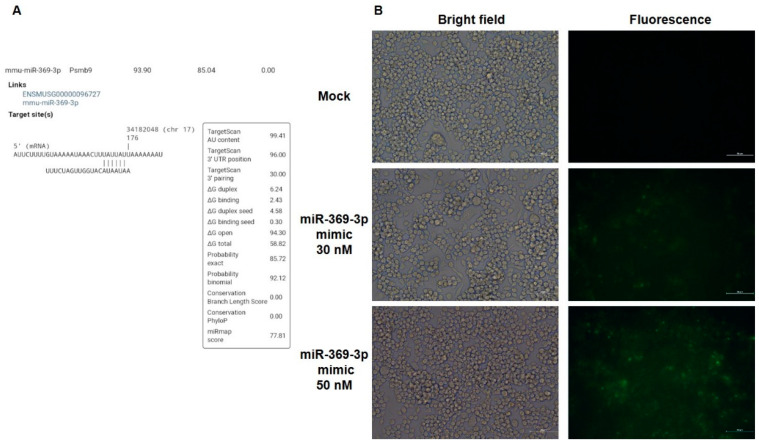
miR-369-3p targets Psmb9, binding a specific sequence in 3′UTR mRNA. (**A**) Sequence alignment of the miR-369-3p base pairing sites in 3′ UTR of Psmb9 mRNA. (**B**) Transfection efficiency of RAW 264.7 mouse macrophages. RAW 264.7 cells were transfected with FAM-labeled miR-369-3p mimic at concentrations of 30nM and 50nM. Representative images of RAW 264.7 transfected with FAM-labeled miR-369-3p mimic. Bright field (left) and fluorescence (right) images for the mock condition and transfected conditions were acquired by fluorescence microscopy. Original magnification, ×20. Scale bar presents 50 μm.

In our previous works [30,31], we reported that miR-369-3p was able to modulate chronic inflammation, regulating C/EBP-β and NOS2. To study further molecular targets of miR-369-3p, we performed a bioinformatic analysis to predict other potential miR-369-3p target genes. We found that Psmb9 was a potential target gene of miR-369-3p, and encodes for an immunoproteasome subunit (Figure 1A). Moreover, alignment of the Psmb9 mRNA sequence from Homo sapiens confirmed that the binding site of miR-369-3p within the Psmb9 3′UTR was highly conserved.

### 2.2. miR-369-3p Directly Decreased PSMB9 Expression

**Figure 2:**
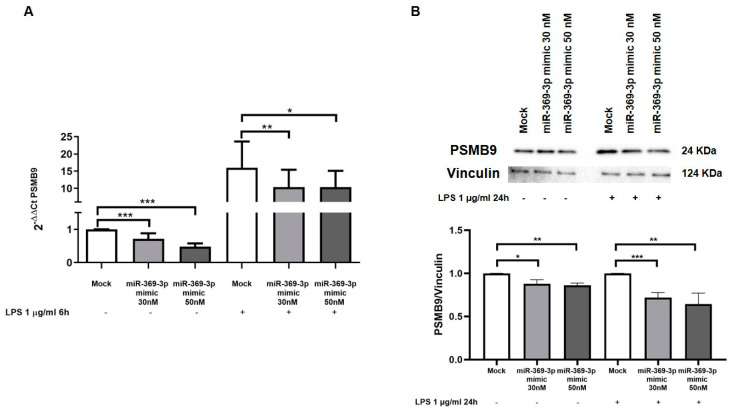
Modulation of PSMB9 mRNA and protein expression by miR-369-3p in RAW264.7 cells. (**A**) The mRNA expression levels of PSMB9 were evaluated by qRT-PCR in RAW264.7 cells transfected with 30 nM and 50 nM of miR-369-3p mimic, both unstimulated and LPS-stimulated. (**B**) Western blot analysis of PSMB9 protein expression after miR-369-3p mimic transfection in the RAW264.7 cell line, both unstimulated and LPS-stimulated. miR-369-3p transfection led to a significant decrease in PSMB9 in LPS-unstimulated as well LPS-stimulated samples. Vinculin was used as housekeeping protein to normalize the data. Data were obtained by comparing normalized sample values to the normalized mock sample value (* *p* < 0.05; ** *p* < 0.01; *** *p* < 0.001).

To functionally confirm the results of bioinformatic analysis, we carried out transient transfection with molecules of miR-369-3p mimic in the macrophage RAW264.7 cell line. Firstly, we examined the efficacy of transfection using a fluorescence-labeled miR-369-3p mimic (Figure 1B). Then, we investigated the effect of transfection on the expression levels of the Psmb9 gene. Real time-PCR revealed that transient transfection with the miR-369-3p mimic at 30 nM and 50 nM concentrations significantly decreased Psmb9 mRNA expression (*p* < 0.001; Figure 2A).

LPS is a potent inducer of immune response and has been demonstrated to augment the expression of immunoproteasome subunits in RAW264.7 [14,32]. Therefore, we examined whether the increased expression of miR-369-3p was able to reduce Psmb9 mRNA also after LPS stimulation. We found that, after LPS stimulation, in mock conditions Psmb9 mRNA expression was significantly increased; however, raising the amount of intracellular miR-369-3p to both 30 nM and 50 nM concentrations significantly decreased the mRNA expression of Psmb9 (*p* < 0.05; Figure 2A).

Western blot analysis confirmed that miR-369-3p was able to reduce PSMB9 protein expression both in unstimulated and stimulated conditions. In steady-state cells, the protein expression levels were evaluated following transfection with the miR-369-3p mimic. As shown in Figure 2B, transient transfection led to a substantial reduction in PSMB9 protein levels compared to those in mock control (*p* < 0.05; Figure 2B). Moreover, after LPS stimulation, Western blot analysis evidenced a correspondingly increased protein expression. Additionally, in this case, the transfection of miR-369-3p mimic at both concentrations notably decreased the expression of the target protein (*p* < 0.01; *p* < 0.001; Figure 2B).

### 2.3. miR-369-3p Indirectly Regulated Components of the Immunoproteasome Complex

In addition to the β1i subunit (PSMB9), the inner beta rings of immunoproteasome are composed of two other subunits, namely β5i and β2i, also known as PSMB8 and PSMB10, respectively. The three immune subunits interact to promote the immunoproteasome assembly to carry out the catalytic activities [9,13,15].

**Figure 3:**
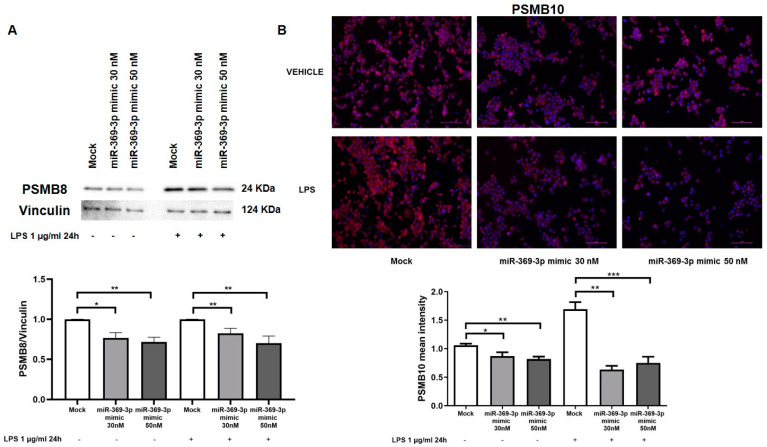
Modulation of PSMB8 and PSMB10 protein expression by miR-369-3p in RAW264.7 cells. (**A**) Western blot analysis of PSMB8 protein expression after miR-369-3p mimic transfection in RAW264.7 cell line unstimulated and LPS-stimulated. The miR-369-3p transfection led to a significant decrease in PSMB8 in LPS-unstimulated as well LPS-stimulated samples. Vinculin was used as housekeeping protein to normalize the data. Data were obtained by comparing normalized sample values to the normalized mock sample value. (**B**) Immunofluorescence staining of PSMB10 in RAW264.7 cell cultures after miR-369-3p mimic transfection. PSMB10 mean intensity was quantified on nuclei number as the mean of three images per cell slide. Original magnification, ×20. Scale bar presents 50 μm. * *p* < 0.05; ** *p* < 0.01; *** *p* < 0.001.

In order to verify whether the reduction in PSMB9 by miR-369-3p caused a decrease in the PSMB8 and PSMB10 in RAW264.7 macrophages, the protein expression of PSMB8 was investigated by Western blot analysis. As displayed by blot images and histograms, the protein expression of PSMB8 was decreased after transfection with miR-369-3p mimic (*p* < 0.05; Figure 3A). Moreover, although stimulation with LPS led to a considerably increased PSMB8 expression in mock samples, miR-369-3p significantly decreased the expression of this subunit at both concentrations (*p* < 0.01; Figure 3A).

Immunofluorescence images showed the expression of PSMB10 in RAW264.7 cell cultures. As shown in Figure 3B, PSMB10 is localised in the cytoplasm. The images showed that after transient transfection with the miR-369-3p mimic, PSMB10 expression significantly decreased compared with the mock control in both basal conditions and under LPS-stimulated conditions (*p* < 0.01; Figure 3B).

### 2.4. PA28α and PA28β Subunits Were Decreased by miR-369-3p

**Figure 4:**
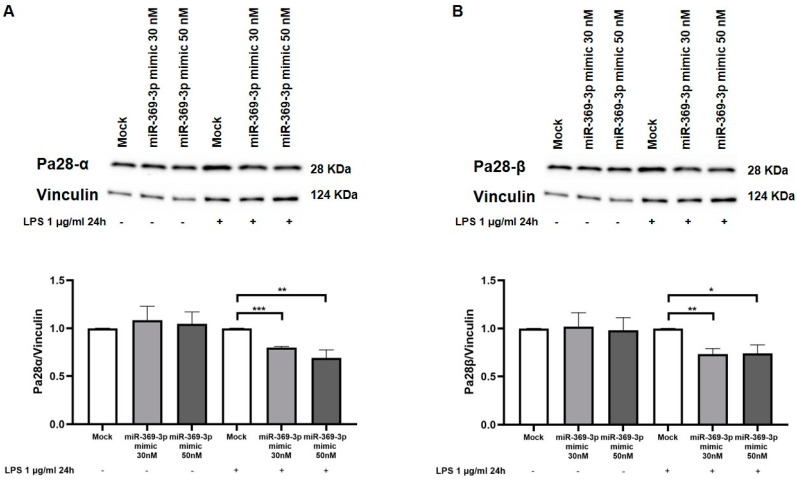
PA28α and PA28β protein expression modulated by miR-369-3p in RAW264.7 cells. Western blot analysis of PA28α (**A**) and PA28β (**B**) proteins after miR-369-3p mimic transfection in the RAW264.7 cell line, both unstimulated and LPS-stimulated. In basal conditions, miR-369-3p transfection did not influence the expression of these proteins. In LPS-stimulated cells, the miR-369-3p transfection resulted in a significant reduction in PA28α and PA28β. Vinculin was used as housekeeping protein to normalize the data. Data were obtained by comparing normalized sample values to the normalized mock sample value (* *p* < 0.05; ** *p* < 0.01; *** *p* < 0.001).

PA28 is a regulatory complex consisting of the PA28α and PA28β subunits, which form a cap that interacts with the 20s core complex through external α-rings. PA28 regulates the catalytic activities of iP subunits and plays a key role in the degradation of damaged proteins and in processing the antigenic peptides for presentation to the MHC-I [13]. Consequently, we investigated whether the PA28 subunits were influenced by transfection with miR-369-3p mimic. As shown from protein expression analysis, in basal conditions, neither subunit underwent any variation. The activation of the immune response via TLR signalling led to an increase in PA28 subunit expression, so we investigated the expression of PA28α and PA28β proteins after stimulation with LPS. Our data showed that the expression of these two subunits increased after LPS stimulation as compared to both mock unstimulated and mock LPS-stimulated conditions. Even then, the transfection with miR-369-3p mimic significantly reduced the expression of PA28α and PA28β (*p* < 0.05; Figure 4A,B). These results suggest that the modulation of the iP catalytic core subunits by miR-369-3p could indirectly influence the recruitment of the PA28 complex.

### 2.5. miR-369-3p Reduced Immunoproteasome Activities

**Figure 5:**
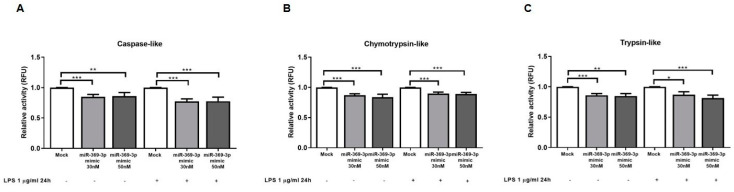
miR-369-3p reduced immunoproteasome activities. RAW264.7 cells transfected with miR-369-3p mimic at 30 nM or 50 nM concentrations showed decreased caspase-like (PSMB9) (**A**), Chymotrypsin (PSMB8) (**B**) and Trypsin (PSMB10) (**C**) activities, with and without LPS stimulation. * *p* < 0.01; ** *p* < 0.001; *** *p* < 0.0001.

PSMB9, PSMB8 and PSMB10 are responsible for the caspase-like, chymotrypsin-like and trypsin-like activities, respectively, of the immunoproteasome. To confirm the proteasome inhibition by miR-369-3p transfection, we evaluated the effect of miR-369-3p mimic on immunoproteasome activities in RAW264.7 cells. As shown in Figure 5, miR-369-3p significantly decreased the activity of the immunoproteasome subunits in macrophages. Specifically, a significant reduction in caspase-like activity (PSMB9) was observed in RAW264.7 cells transfected with miR-369-3p mimic at 30 nM or 50 nM concentrations as compared to mock control (*p* < 0.001; Figure 5A). miR-369-3p mimic also attenuated the Chymotrypsin and Trypsin activities (*p* < 0.01, Figure 5B,C).

Since the stimulation with LPS of macrophages has been reported to induce the formation of immunoproteasomes and to trigger the enzymatic activities of LMP subunits [14], we likewise examined the immunoproteasome activities after transfection with miR-369-3p in LPS-stimulated RAW264.7 cells. Additionally, in this case, transfection with miR-369-3p mimic had a positive effect on RAW264.7 LPS-stimulated cells, significantly reducing the caspase-like, chymotrypsin-like and trypsin-like activities (*p* < 0.05, Figure 5A–C).

### 2.6. PSMB9 Expression in IBD Patients

To further confirm our results and to highlight that PSMB9 is a good target for the treatment in IBD, we examined its mRNA expression from a dataset downloaded from the Gene Expression Omnibus database (GSE6731). This dataset consists of gene expression profiles of active and inactive areas of IBD patients and healthy controls [33].

**Figure 6:**
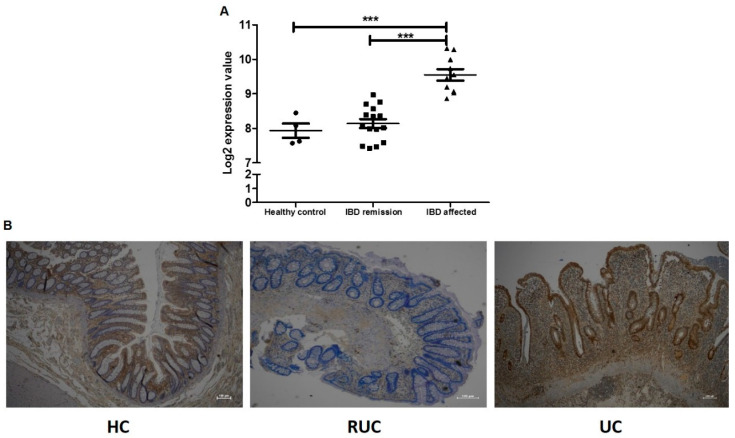
PSMB9 expression in IBD patients. (**A**) Analysis of colonic tissue from IBD patients and controls downloaded from the GEO database (GSE6731). Mean expression data were expressed as log of expression values. (**B**) PSMB9 protein expression at IHC in formalin-fixed, paraffin-embedded tissues obtained from healthy controls (HC), remission UC (RUC) patients and active ulcerative colitis (UC) patients. Original magnification, ×4. Scale bar presents 100 μm. *** *p* < 0.0001.

We evaluated the different gene expression of PSMB9 in IBD affected and unaffected patients. We found that PSMB9 expression was significantly increased in the acute IBD group compared to the remission IBD group (*p* < 0.0001) and control group (*p* < 0.0001) (Figure 6A).

To confirm these data, we evaluated PSMB9 protein expression on tissue specimens of a cohort of ulcerative colitis (UC) patients and healthy controls (HC) collected at our institute. We found that in acute UC patients, PSMB9 showed a strong and diffuse positivity in the surface epithelial cells and in the crypts, areas of structural distortions induced by chronic inflammation (Figure 6B).

Instead, in remission UC (RUC) patients, PSMB9 expression was absent both in epithelial and stromal compartments. We observed an optimal renewal of mucin and glandular components of intestinal epithelium, although crypts still presented a structural distortion with a slight gap between the base of the crypts and muscularis mucosa (Figure 6B).

In HC, the staining revealed that PSMB9 was not expressed on the epithelium surface or crypts and glands of the normal mucosa. An unspecific positivity was observed in the lymphoplasmacellular component of the lamina propria (Figure 6B).

These data confirm that the PSMB9 increase was closely related to the disease state of IBD patients.

## 3. Discussion

Macrophages are present in the intestinal mucosa, contributing to maintaining intestinal homeostasis. Their altered activation could be involved in the onset of abnormal inflammatory responses and they have been shown to have a crucial role in the pathogenesis of IBD. In the intestine, several works have demonstrated that the macrophages are able to massively infiltrate the mucosa, triggering an aberrant inflammatory response that alters the integrity of the intestinal barrier [34,35]. Moreover, they are also responsible for increased levels of TNF-α, IFN-γ and other proinflammatory cytokines that are well-known to be present in patients affected by IBD [36].

The proteasome complex is ubiquitously expressed in all cell types and plays an important role in regulation of the normal cell cycle, differentiation and cell death mechanisms. In immune cells, the inflammatory environment induces replacement of the constitutive proteasome with immunoproteasome [10,11,12,13,14]. During immune responses, the immunoproteasome is able to process abnormal proteins, generating the antigen peptides under inflammatory and stress stimuli [13,15]. Previous studies showed that in IBD patients, the expression of immunoproteasome subunits was increased, demonstrating its potential role in the IBD pathogenesis [18,19,20].

The inhibition of immunoproteasome may be a good targeted treatment of several diseases including cancer, autoimmune diseases and IBD [21,23,24,37,38]. Various studies have identified several immunoproteasome inhibitors that act at many different levels. The compounds discovered include a selective inhibitor for one of the proteasome subunits [22,37,39], as well as compounds that inhibit the NF-kB signaling pathway activated by the immunoproteasome process [24,38].

miRNAs act as gene regulators, inhibiting the protein expression working at transcription levels; they may be used for therapeutic purposes. However, the involvement of miRNAs in regulating immunoproteasome activities is still poorly understood. In chronic HCV infection, the involvement of miR-125b in the regulation of PSMB9 expression was discovered [40]. miR-451a, identified as a regulator of the progression of several cancers, was investigated in association with PSMB8 in prostate cancer. This subunit was closely associated with tumor invasion, metastasis, and poor survival. miR-451a functionally targets PSMB8, reducing its expression, and consequently decreased cell proliferation, invasion and promoted apoptosis [41].

In the present study, we identified PSMB9 as a putative target of miR-369-3p. In our previous works, we demonstrated that miR-369-3p reduced the LPS-induced inflammatory response, and regulated the expression levels of NOS2, decreasing the production of pro-inflammatory agents in dendritic cells [30,31]. In this study, we found that the levels of PSMB9 mRNA and protein were reduced after miRNA transfection. Moreover, this regulation by miR-369-3p caused an indirect modulation of the other immunoproteasome components.

As previously described, in vitro LPS stimulation augments the expression of immunoproteasome subunits, triggering their activation [14,32]. Here, we demonstrated that also under inflammatory conditions, miR-369-3p was able to obstruct the expression of PSMB9 as well as of other immunoproteasome members of catalytic core. In addition, raising the intracellular levels of miR-369-3p, the caspase-like, trypsin-like and chymotrypsin-like activities were found to be evidently decreased in response to LPS. During inflammatory conditions, PA28 complexes, immunoproteasome activators, are upregulated. The binding of PA28 complexes with the immunoproteasome nucleus improved the catalytic function of the immunoproteasome. Here, we have shown that PSMB9 modulation by Mir-369-3p indirectly decreased the expression of PA28 subunits in inflammatory conditions. To date, the mechanisms underlying this correlation are little known. Future studies may allow a greater understanding of these processes.

The decrease in miR-369-3p levels in IBD human biopsies has been demonstrated [31]. Now, we confirm that PSMB9 expression was increased in the acute IBD compared to the healthy group, remaining at low levels in remission IBD. This validates the altered expression of PSMB9, and reinforces the concept of PSMB9 as a potential target for a therapeutic approach [33].

Several studies were based on the identification and development of proteasome inhibitors applicable as potential therapy for different diseases [17,37]. However, in some instances, the use of the single molecule could be insufficient to reduce the inflammatory state [23,42]. Here, we demonstrate that the use of miR-369-3p alone is able to inhibit the entire immunoproteasome complex. This offers a major advantage since a single molecule can inhibit the multi-enzymatic complex, thus further supporting the idea of its possible application in therapy.

This work is a preliminary study aimed at demonstrating modulation of the immunoproteasome by miR-369-3p. Future studies will be needed to confirm the effect of miR-369-3p administration in IBD mice models.

In conclusion, we demonstrate that miR-369-3p regulating immunoproteasome complex are able to modulate the inflammatory state of patients affected by IBD. Thus, we suppose that the induction of miR-369-3p could ameliorate intestinal inflammation. Our findings may shed light on new therapeutic strategies for IBD.

## 4. Materials and Methods

### 4.1. Cells Culture

RAW 264.7 mouse macrophages were obtained from ATCC (American Type Culture Collection, Manassas, VA, USA). The cultured cells were maintained in Dulbecco’s Modified Eagle Medium (DMEM, Thermo Fisher Scientific, Waltham, MA, USA), containing 10% heat-inactivated Fetal Bovine Serum (FBS, Thermo Fisher Scientific, Waltham, MA, USA), 1% 10,000 µg/mL streptomycin and 10,000 U/mL penicillin (Thermo Fisher Scientific, Waltham, MA, USA), 1% 1M HEPES (Sigma-Aldrich, St. Louis, MO, USA) and 1% 100 mM sodium pyruvate (Sigma-Aldrich, St. Louis, MO, USA). Cells were grown at 37 °C in a humidified atmosphere with 5% CO_2_.

### 4.2. miR-369-3p Mimic Transfection

RAW 264.7 cell lines were seeded into 12-well plates at a density of 8 × 10^5^ cells/well (for RNA and protein extraction), 1.5 × 10^5^ cells/well in Lab-Tek Chamber Slides (for immunofluorescence assays), and in 96-well plates at a density of 5 × 10^5^ cells/well (for immunoproteasome activity assays). Cells were transfected with miR-369-3p mimic (Qiagen, Hilden, Germany) at a concentration of 30 nM and 50 nM using TransIT-TKO Transfection Reagent (Mirus Bio LLC, Madison, WI, USA) according to the manufacturer’s instructions. Each experiment was normalized with mock control, corresponding to cells transfected with empty liposome. Cell cultures were then stimulated with LPS (Sigma-Aldrich, St. Louis, MO, USA) at a final concentration of 1 μg/mL and incubated for an additional 6 h for RNA isolation and 24 h for proteins extraction.

### 4.3. RNA Extraction and Real-Time PCR

Cells were lysed with TRIzol reagent (Invitrogen, Carlsbad, CA, USA) and, based on the manufacturer’s protocol, total RNA was extracted and then resuspended in ribonuclease-free water. The concentration of RNAs was determined through the NanoDrop Spectrophotometer (Thermo Fisher Scientific, Waltham, MA, USA). cDNAs were synthesized from 1 μg of total RNAs using iScript Reverse Transcription Supermix (BioRad Laboratories, Hercules, CA, USA) according to the manufacturer’s instructions. qPCR was conducted on a CFX96 System (Biorad Laboratories, Hercules, CA, USA). The expression levels of the target gene and housekeeping gene were measured using SsoAdvanced Universal SYBR green PCR Supermix (BioRad Laboratories, Hercules, CA, USA) and QuantiTect Primer Assay for Psmb9 and Gapdh (Qiagen, Hilden, Germany). Gapdh gene amplification was used as the housekeeping control to normalize the gene expression of Psmb9.

### 4.4. Protein Isolation and Western Blot Analysis

For proteins’ isolation, cells were lysed with T-PER Protein Extraction Reagent (Thermo Fisher Scientific, Waltham, MA, USA) adding protease inhibitor cocktail (Sigma-Aldrich, St. Louis, MO, USA). Protein concentrations were determined using Bradford’s protein assay (Biorad Laboratories, Hercules, CA, USA). For each protein lysate, 20 μg of total proteins were separated in 4–20% Mini-PROTEAN TGX Stain-Free Protein Gels (Biorad Laboratories, Hercules, CA, USA) and blotted on Trans-Blot Turbo Mini 0.2 µm PVDF membranes (Biorad Laboratories, Hercules, CA, USA). For protein detection, PVDF membranes were incubated in iBind automated Western Systems (Thermo Fisher Scientific, Waltham, MA, USA) following the manufacturer’s instructions. Images containing bands were captured using the Chemidoc System (Biorad Laboratories, Hercules, CA, USA) setting the acquisition protocol on the automatic exposure of Image lab Software (Biorad Laboratories, Hercules, CA, USA) and quantified by the Image J program. The protein expression was normalized to Vinculin housekeeping protein expression.

The primary antibodies used were rabbit pAb PSMB9 (Invitrogen, #PA1-1960), rabbit mAb PSMB8/LMP7 (Cell Signaling, Technology, Danvers, MA, USA, #13635), rabbit mAb Pa28α (Cell Signaling, Technology, Danvers, MA, USA, #9643), rabbit mAb Pa28β (Cell Signaling, Technology, Danvers, MA, USA, #2409), and rabbit mAb Vinculin (Cell Signaling, Technology, Danvers, MA, USA, #13901). The secondary antibodies, horseradish-peroxidase-conjugated, consisted of Goat Anti-rabbit IgG-(H + L)-HRP conjugate (Invitrogen, Carlsbad, CA, USA, #31466).

### 4.5. Immunofluorescence

Cells were washed three times with PBS and fixed with 4% paraformaldehyde (PFA, Sigma-Aldrich, St. Louis, MO, USA) for 15 min at room temperature. Then, they were washed twice with PBS and blocked in PBS + BSA 3% and Triton-X 0.1% for 1.5 h at room temperature. Samples were incubated in primary antibody rabbit pAb PSMB10 (Invitrogen, PA5-96327) for 3 h at room temperature. After washing with PBS, cells were then incubated with secondary antibody chicken anti-Rabbit IgG (H + L) Alexa Fluor 594 (Invitrogen, A-21442) for 1 h at room temperature in the dark. ProLong Gold Antifade Mountant with DAPI (Thermo Fisher Scientific, Waltham, MA, USA) was applied to each sample and mounted with a glass cover slip. Images were obtained using the fluorescence microscope (Eclipse Ti2, Nikon Inc., Melville, NY, USA). For each sample, 3 images were captured in different positions and staining was quantified using ImageJ analysis software.

### 4.6. Measurement of Immunoproteasome Activity

After the above treatment, the 96-well plates were equilibrated at room temperature for 10 min before adding the reagents to detect immunoproteasome activities. To detect chymotrypsin-like, trypsin-like and caspase-like enzymatic activities, an equal volume of the Proteasome-Glo Cell-Based Reagent (Promega, Madison, WI, USA) was added in each well according to the manufacturer’s instruction. The plates were incubated at room temperature for 10 min in the dark and the relative luminescence units (RLU) of assays were determined in the LUMIstar Omega microplate luminometer (BMG Labtech, Ortenberg, Germany) according to the manufacturer’s directions.

### 4.7. UC Specimens and Immunohistochemistry (IHC)

Formalin-fixed and paraffin-embedded tissue blocks were obtained from 28 patients (20 men and 8 women) subdivided into three groups: the HC group (healthy control, n = 10 patients) consisting of intestinal surgical resections from patients without ulcerative colitis (UC); the acute UC group (n = 10 patients, mean age 61 years, and range 33–80) including total or subtotal proctocolectomies from patients with longstanding ulcerative colitis refractory to medical therapies; and the remission UC group (RUC, n = 8 patients, mean age 45 years, and range 24–57) including biopsy samples from quiescent ulcerative colitis patients.

Sections stained with hematoxylin and eosin were reviewed by a pathologist to confirm the adequacy of the sample and to evaluate the morphologic and/or pathological characteristics of the samples. For IHC detection, 4 µm sections were cut and mounted on Apex Bond IHC slides (Leica Biosystems, Buffalo Grove, IL, USA). IHC staining procedures were performed on the BOND III automated immunostainer (Leica Biosystems, Buffalo Grove, IL, USA), from deparaffinization to counterstaining with hematoxylin. Tissue sections were incubated with an anti-PSMB9 primary antibody (MAb OTI1C4, Invitrogen, Waltham, MA, USA, 1:4000 dilution) for 30 min at room temperature. The Bond Polymer Refine Detection Kit (Leica Biosystems, Buffalo Grove, IL, USA) was used as a visualization and chromogen reagent according to the manufacturer’s instructions. Samples were recorded as negative when the number of stained cells was less than 5%.

### 4.8. Bioinformatics and Statistical Analysis

Putative miRNA gene targets were predicted by miRmap (https://mirmap.ezlab.org/ (accessed on 15 December 2021)) [43] algorithm.

Data were analyzed using GraphPad Prism software and expressed as the mean  ±  SEM. Statistical significance of data resulting from different conditions was examined with two-tailed Student’s *t* test. Data derive from at least three independent experiments. Differences among experimental conditions were considered statistically significant at *p* < 0.05.

## 5. Patents

An Italian patent entitled “Pharmaceutical composition based on miR-369-3p as active ingredient for the treatment of chronic inflammatory disorders” (patent n° 102018000007954) was issued on 3 August 2020 to the Ente Ospedaliero Specializzato in Gastroenterologia “Saverio de Bellis”.

## Data Availability

Not applicable.
